# Nonpharmacologic, nonherbal management of menopause-associated vasomotor symptoms: an umbrella systematic review (protocol)

**DOI:** 10.1186/s13643-016-0232-6

**Published:** 2016-04-07

**Authors:** Karen M. Goldstein, Jennifer R. McDuffie, Megan Shepherd-Banigan, Deanna Befus, Remy R. Coeytaux, Megan G. Van Noord, Adam P. Goode, Varsha Masilamani, Soheir Adam, Avishek Nagi, John W. Williams

**Affiliations:** Center for Health Services Research in Primary Care, Durham Veterans Affairs Medical Center, Durham, NC USA; Department of Medicine, Division of General Internal Medicine, Duke University Medical Center, 411 W. Chapel Hill Street; Suite 500, Durham, NC 27701 USA; Duke University School of Nursing, Durham, NC USA; Duke Clinical Research Institute, Durham, NC USA; Duke University Medical Center Library, Durham, NC USA; Department of Physical Therapy, Duke University Medical Center, Durham, NC USA; Department of Medicine, Duke University Medical Center, Durham, NC USA

**Keywords:** Vasomotor symptoms, Menopause, Nonpharmacologic therapy, Umbrella review

## Abstract

**Background:**

Vasomotor symptoms such as hot flashes and night sweats are a common concern of perimenopausal and postmenopausal women and are associated with a decreased quality of life. These symptoms can be effectively managed with hormone therapy, but safety concerns limit its use. Thus, understanding the effectiveness of nonpharmacologic therapies such as acupuncture or yoga is critical to managing these common symptoms in older women. Our review seeks to address the following question: In women with menopause-associated vasomotor symptoms, what are the effects on health-related quality of life, vasomotor symptoms, and adverse events of the following nonpharmacologic, nonherbal interventions as compared with any inactive control or active comparator: (a) acupuncture, (b) yoga, tai chi, and qigong, (c) structured exercise, and (d) meditation, mindfulness-based practices, and relaxation?

**Methods:**

We describe a protocol for an umbrella review approach, supplemented by evaluating randomized controlled trials (RCTs) published after the most recent good-quality systematic review for each of the eligible interventions. Specific interventions were chosen based on current literature and with input from a technical expert panel and organizational stakeholders. We will conduct a thorough literature search and perform a quality assessment of potentially included systematic reviews and RCTs.

**Discussion:**

Our umbrella review, supplemented by an additional search for eligible RCTs, aims to synthesize existing evidence on the use of nonpharmacologic, nonherbal interventions to manage bothersome vasomotor symptoms in perimenopausal and postmenopausal women.

**Systematic review registration:**

PROSPERO CRD42016029335

**Electronic supplementary material:**

The online version of this article (doi:10.1186/s13643-016-0232-6) contains supplementary material, which is available to authorized users.

## Background

Hot flashes and night sweats (vasomotor symptoms or VMS) are two of the most common symptoms reported by perimenopausal and postmenopausal women and are experienced by as many as 80 % of women [1]. The mean age at VMS onset is 51 years, and frequent VMS can last more than 7 years [2], leading to increased healthcare encounters for symptom relief [3] and reductions in quality of life (QOL) [4, 5]. The impact of VMS on QOL can be worse for certain populations of women such as those who undergo surgical rather than natural menopause [6]. Moreover, the degree to which VMS are bothersome is determined not only by how frequently the symptoms occur but also by other factors such as duration of VMS and coexistent sleep problems [7]. Hormone therapy is an effective treatment for reducing VMS but is recommended only for short-term use due to substantial risks, such as cardiovascular events and uterine and breast cancers [8, 9]. Also, some women are not candidates for hormone therapy because of comorbid health conditions such as liver disease or a history of blood clots. This situation leaves women with VMS in need of nonhormonal treatment options for many years. For this reason, the identification of safe and effective nonhormonal strategies is needed.

Many perimenopausal and postmenopausal women are already using nonpharmacologic agents to manage VMS [10–12]. Nonpharmacologic treatments for VMS include herbal remedies (e.g., black cohosh), mind and body practices (e.g., yoga, tai chi), structured exercise programs, and complementary and alternative medicine interventions (e.g., acupuncture) [13]. A 2015 Agency for Healthcare Research and Quality (AHRQ) systematic review [14] examined the comparative effectiveness of estrogens, isoflavones, selective serotonin reuptake inhibitors (SSRIs) and serotonin-norepinephrine reuptake inhibitors (SNRIs), gabapentin, black cohosh, and ginseng for menopausal symptoms, including VMS. However, the AHRQ review did not address nonpharmacologic, nonherbal interventions such as mind and body practices, structured exercise programs, or complementary and alternative medicine interventions. Also in 2015, the North America Menopause Society released a position statement providing recommendations for many such intervention types and graded the level of evidence for their recommendations; however, this was not a formal systematic review of the literature and included a discussion of both herbal and other nonhormonal pharmacologic treatments [15].

Because nonpharmacologic treatments are conceptually attractive to treat menopause-associated VMS, we will evaluate evidence from systematic reviews (SRs) and randomized controlled trials (RCTs) on selected nonpharmacologic, nonherbal therapies. Our objective with this review is to summarize and update the evidence from SRs and recent RCTs on selected nonpharmacologic and nonherbal approaches for the management of menopause-associated VMS. Specifically, we seek to answer the following key question: In women with VMS that are associated with perimenopause or postmenopause, what are the effects on health-related quality of life, VMS, and adverse events of the following nonpharmacologic, nonherbal interventions as compared with any inactive control or active comparator: (1) acupuncture, (2) yoga, tai chi, and qigong, (3) structured exercise, and (4) meditation, mindfulness-based practices, and relaxation?

## Methods/design

This protocol has been registered in the PROSPERO database for systematic reviews, a web-based international registry of systematic review protocols (#CRD42016029335) [16]. A PRISMA-P checklist was completed for this protocol (Additional file 1).

Given the multiple high-quality SRs in this topic area, we decided to use a method commonly known as an umbrella review or a review of reviews. Additionally, we will supplement this approach by searching for and evaluating RCTs published after the most recent good-quality SR for each of the eligible interventions. We will follow methodological guidance from the Cochrane Collaboration [17] and AHRQ’s Evidence-based Practice Centers [18].

## Selection of nonpharmacologic interventions

Numerous interventions could be considered nonpharmacologic treatments for VMS in perimenopausal and menopausal women, particularly variations of complementary and alternative medicine approaches to symptom control [10]. To focus the selection of interventions for this review, we invited individuals with expertise in the field of menopause management both from within the Veterans Health Administration (VHA) and outside the VHA to participate in a technical expert panel. This panel provided consultation during the process of reviewing and organizing a list of potential interventions originally generated by the primary review team based on published literature and clinical practice. Final selection of interventions for review (Table 1) was chosen based on priorities from VHA stakeholders who proposed and supported this review. Of note, we acknowledge that some included interventions (e.g., yoga) could potentially fit in multiple categories (e.g., structured exercise, meditation) and that some interventions are often delivered together in practice. As our understanding of the literature develops, we may add or change intervention categories as appropriate. To enable our ability to identify meaningful and specific intervention treatment effects, eligible interventions that are part of a multimodal intervention will be included only if it is possible to isolate the effect of the eligible intervention. If multimodal interventions are included in relevant SRs, we will prioritize results that isolate the effects of the eligible intervention.

**Table 1:** Eligible interventions and definitions

Intervention category	Definitions and examples
Acupuncture, acupressure	Acupuncture from any tradition will be considered, including auricular acupuncture, electroacupuncture, acupressure, and laser acupuncture. We will exclude studies where acupuncture was administered in conjunction with Chinese herbal therapies. Cupping therapy will be excluded unless it is simply a component of an acupuncture intervention.
Yoga, tai chi, qigong (as defined by study investigators)	Yoga is generally defined as physical exercises and bodily positions or postures, breathing control practices, and meditation.
	Tai chi is generally defined as series of movements performed in a slow, focused manner accompanied by deep breathing.
	Qigong is generally defined as system of coordinated body posture and movement, breathing, and meditation.
Meditation, mindfulness, relaxation	Practices include: • Mindfulness-based stress reduction • Progressive relaxation • Bernstein and Borkovec’s progressive relaxation • Everly and Rosenfeld’s passive relaxation • Madder’s applied relaxation • Poppen’s behavioral relaxation training • Mitchell method • Alexander technique • Benson’s relaxation response • Guided imagery-based approaches • Roll breathing • Paced respiration • 4-7-8 breathing technique • Hypnosis • Other approaches that focus on diaphragmatic breathing
Structured exercise, physical activity	Defined as physical activity that is regular and done with the intention of improving or maintaining physical fitness or health or performed as part of a class or with support from a health professional.

## Eligible SRs and RCTs

We will include SRs and RCTs that evaluate an eligible intervention for bothersome VMS associated with perimenopause or postmenopause. Perimenopause is defined as amenorrhea for greater than 60 days in the past 12 months; postmenopause is defined as being without a menstrual cycle due to spontaneous or surgical reasons for the preceding 12 months [19, 20]. For the SRs, we will accept the definition of VMS as used by the authors and will track any variations in what was used. For RCTs, we will consider bothersome VMS defined as any of the following: self-identified “bothersome” hot flashes, moderate to severe vasomotor symptoms as defined by the FDA [21], hot flashes that occur at least 6 days in the previous 2 weeks [2], or hot flashes that are associated with functional impairment (e.g., impairment in role, social, emotional, or physical functioning). Women with breast cancer will be included. Eligible interventions are listed in Table 1. Specifically, we will include interventions that fall under four main categories: acupuncture, acupressure; yoga, tai chi, qigong; structured physical activity; and meditation, mindfulness, relaxation. Because the interventions in each of these categories have multiple subtypes, we have listed those that we believe will be relevant to the objective of this review based on current literature and expert opinion. Additionally, we have provided definitions for the intervention types of interest to further guide inclusion (e.g., structured exercise). Any inactive control (e.g., waitlist, attention control, sham acupuncture, information control, or unenhanced usual care) or active comparator (e.g., hormone treatments, antidepressants, dietary supplements, health education, unstructured forms of exercise) will be allowed. SRs that include mixed settings that are inclusive of outpatient/community settings will be eligible, but we will prioritize results from outpatient/community settings. If it is impossible to disaggregate results, we will describe the number of studies/patients contributed by studies conducted in outpatient/community settings. For SRs, we will accept the timing of outcome assessments as specified by the review. For RCTs, outcomes must be assessed ≥60 days after treatment assignment in order to provide findings based on a reasonably long duration of follow-up given the chronicity of VMS; the choice of this duration cutoff was vetted with our technical expert panel. Additionally for inclusion, SRs and RCTs must include at least one of our primary or secondary outcomes of interest as described in the following section.

We define SRs as studies that include an explicit search, prespecified eligibility criteria, an evaluation of the quality of included studies or risk of bias, and a synthesis or an attempt to synthesize findings quantitatively and/or qualitatively [22]. We will exclude SRs that review complementary and alternative therapies in general without a specific focus on an intervention of interest. We will also exclude SRs that are found to be of poor quality after quality assessment is completed (see “Assessment of methodological quality of SRs” section below). RCTs will not be excluded due to quality limitations, but we will make interpretations that incorporate our risk of bias assessment.

## Review outcomes

We have two primary outcomes for this study: (1) overall and condition-specific health-related quality of life (HRQOL) and (2) frequency and intensity of VMS. HRQOL was chosen as one of the primary outcomes for this study because it constitutes a global measure of the impact of VMS on an individual’s well-being and because of the interest of our VHA stakeholders and existing evidence that women Veterans may experience greater adverse effects on QOL from VMS than non-Veteran women [23]. We will assess this outcome based on the measures available from included studies and reviews that may be specific to the menopausal condition (e.g., a menopause rating scale [6] or the Women’s Health Questionnaire [24]) or more general. Secondary outcomes include psychological well-being (i.e., depressive or anxiety symptoms), sleep quality, and adverse effects. For SRs, we will accept the timing of outcome assessments as specified by the review. For eligible RCTs that are published subsequent to the relevant SR, outcomes must be assessed greater than 60 days after treatment assignment for reasons stated above. We will prioritize validated scales over unvalidated scales or single-symptom measures (e.g., frequency of hot flashes). We also plan to collect information about serious adverse effects [25].

## Literature search methods

We will conduct a search of electronic databases using MeSH keywords and selected free-text terms for the interventions and health conditions of interest as well as terms for SRs and RCTs (Table 2). To ensure completeness, search strategies (Table 3) will be developed in consultation with a master librarian. The search for SRs will be conducted in PubMed and the Cochrane Database of Systematic Reviews. If we identify no SRs for any of the included interventions, we will search PROSPERO (a database of review protocols) [26] for submitted protocols related to the specified health conditions and which may become available to summarize a topic area in the near future. We will include moderate- and high-quality SRs published from January 1, 2010 forward, which will enable us to identify reviews that likely are still current since the median time needed for updating an SR is 5.5 years [14]. Because Cochrane reviews are updated every 2 years and may be published in both the Cochrane database and peer-reviewed journals, we may encounter more than one review published by the same author on the same topic. In this case, we will include only the most recent and fully reported review. We will include only full journal articles (i.e., not abstracts or dissertations). Only English language publications will be included.

**Table 2:** Search concepts and keywords

Concept	MeSH terms	Title/abstract keywords
Menopause	Menopause; Climacteric; Hot Flashes	Menopaus*; Peri-menopaus*; Postmenopaus*; Climacteric*; Hot flash*; Hot flush*; Night sweat*; Vasomotor symptom*
Systematic review	–	Systematic; Systematic Review; Umbrella Review; Meta-analysis
Acupuncture	Acupuncture therapy; Acupuncture; Acupressure	Acupuncture; acupressure; electroacupuncture
Mind-body therapies	Mind-body therapies; Breathing exercises; Imagery (psychotherapy); Meditation; Relaxation therapy; Mind-body relations, metaphysical; Mindfulness, Hypnosis	Mind-body therap*; Mind body medicine; Breathing exercise*; Respiratory muscle training; Guided imagery; Meditation; Relaxation therapy; Relaxation technique*; Alexander technique; Mindfulness-based stress reduction; MBSR; Paced respiration; Alternative medicine, Hypnosis
Yoga, tai chi; qigong	Yoga; Tai Ji	Yoga; Tai Ji; Tai Chi; T'ai Chi; Taiji; Taijiquan; Qi Gong; Qigong; Ch'l Kung; Chi Kung; Kinesiotherapy
Exercise	Exercise; Circuit-based exercise; Muscle stretching exercises; physical conditioning human; Resistance training; Running; jogging; Swimming; Walking; Exercise movement techniques; Sports	Exercise*; Resistance training; Physical activity; Aerobic activity; Sport

**Table 3:** Search strategy. Database: PubMed (all database searches use the same search strategy). Search date: November 9, 2015

Search	Query	Items found
#1	Search "Menopause"[Mesh] OR menopaus*[tiab] OR "Climacteric"[Mesh:NoExp] OR "Hot Flashes"[Mesh] OR peri-menopaus*[tiab] OR perimenopaus*[tiab] OR postmenopaus*[tiab] OR post-menopaus*[tiab] OR climacteric*[tiab] OR hot-flash*[tiab] OR hot flash*[tiab] OR hot-flush*[tiab] OR hot flush*[tiab] OR night sweat*[tiab] OR vasomotor symptom*[tiab]	91,908
#2	Search systematic[sb] OR "Systematic Review"[tiab] OR "Umbrella Review"[tiab] OR meta-analysis[tiab] OR "meta analysis"[tiab]	280,172
#3	Search #1 AND #2	2771
#4	Search "Acupuncture Therapy"[Mesh] OR "Acupuncture"[Mesh] OR "Acupressure"[Mesh] OR "acupuncture"[tiab] OR "acupressure"[tiab] OR "electroacupuncture"[tiab]	23,260
#5	Search #3 AND #4	45
#6	Search "Mind-Body Therapies"[Mesh: NoExp] OR "Mind-Body Therapy"[tiab] OR "Mind Body Therapy"[tiab] OR "Mind-Body Therapies"[tiab] OR "Mind Body Therapies"[tiab] OR "Mind Body Medicine"[tiab] OR "Breathing Exercises"[Mesh] OR "Breathing Exercise"[tiab] OR "Breathing Exercises"[tiab] OR "Respiratory Muscle Training"[tiab] OR "Imagery (Psychotherapy)"[Mesh] OR "Guided Imagery"[tiab] OR "Meditation"[Mesh] OR "Meditation"[tiab] OR "Relaxation Therapy"[Mesh] OR "Relaxation Therapy"[tiab] OR "Relaxation Techniques"[tiab] OR "Relaxation Technique"[tiab] OR "Alexander Technique"[tiab] OR "Mind-Body Relations, Metaphysical"[Mesh] OR "Mindfulness"[Mesh] OR "Mindfulness-based Stress Reduction"[tiab] OR "MBSR"[tiab] OR "paced respiration"[tiab] OR "Hypnosis"[Mesh] OR "Hypnosis"[tiab] OR "Hynotism"[tiab] OR "Hypnotherapy"[tiab] OR "Hynotherapies"[tiab] OR "Mesmerism"[tiab]	16,823
#7	Search #3 AND #6	16
#8	Search "Yoga"[Mesh] OR "Yoga"[tiab] OR "Tai Ji"[Mesh] OR "Tai Ji"[tiab] OR "Tai-ji"[tiab] OR "Tai Chi"[tiab] OR "T'ai Chi"[tiab] OR "Taiji"[tiab] OR "Taijiquan"[tiab] OR "Qi Gong"[tiab] OR "Qigong"[tiab] OR "Ch'I Kung"[tiab]	4440
#9	Search #3 AND #8	22
#10	Search "Exercise"[Mesh:NoExp] OR "Exercise"[Majr] OR "Circuit-Based Exercise"[Mesh] OR "Muscle Stretching Exercises"[Mesh] OR "Physical Conditioning, Human"[Mesh] OR "Resistance Training"[Mesh] OR "Resistance Training"[tiab] OR "Running"[Mesh] OR "Jogging"[Mesh] OR "Swimming"[Mesh] OR "Walking"[Mesh] OR "Exercise"[tiab] OR "Exercises"[tiab] OR "physical activity"[tiab] OR "aerobic activity"[tiab] OR "Exercise Movement Techniques"[Mesh] OR "Sports"[Mesh]	378,950
#11	Search #3 AND #10	204
#12	Search #5 OR #7 OR #9 OR #11	247
#13	Search (#12) AND ("2009/01/01"[Date – Publication] : "3000"[Date – Publication])	132
#14	Search #13 AND "English"[lang]	123

Subsequent to the identification of SRs, we will conduct a search for additional individual RCTs published after the search date noted in the SR for each intervention category. The search for RCTs will be conducted in PubMed, EMBASE, CINAHL, and the Allied and Complementary Medicine Database (AMED). However, if we cannot find any recent SRs on a particular intervention, we will simply report this finding and not try to identify and conduct a de novo synthesis of all primary studies. As with SRs, only English language publications and full journal articles will be included for RCTs.

Using prespecified inclusion/exclusion criteria, titles and abstracts of SRs and RCTs identified through our primary search will be reviewed by two reviewers for potential relevance to the key question. Articles included by either reviewer will undergo full-text screening. At the full-text screening stage, two independent reviewers must agree on a final inclusion/exclusion decision and the rationale for this decision. Articles meeting eligibility criteria will be included for data abstraction. All results will be tracked in both DistillerSR, a web-based data synthesis software program (Evidence Partners Inc., Manotick, ON, Canada), and EndNote^®^ reference management software (Thomson Reuters).

## Assessment of methodological quality of SRs

We will evaluate the quality of SRs in the following manner: the reviewer assigned to abstract the article will perform an initial assessment, which will then be overread by a second reviewer. Disagreements will be resolved between the two reviewers or when needed by arbitration from a third reviewer.

We will use the following key quality criteria for SRs, adapted from ROBIS [27] and AMSTAR [28]: search methods adequate for replication and comprehensive, selection bias avoided, data abstracted reliably, characteristics of primary literature reported and quality assessed appropriately, results synthesized using appropriate methods, publication bias assessed, conflict of interest reported, and conclusions supported by results (Additional file 2). Based on these criteria, SRs will be categorized as good, fair, or poor quality. Good- and fair-quality SRs should provide sufficient information to assess the strength of the body of evidence using the GRADE criteria [29], which includes the major domains of risk of bias, directness, consistency, precision, and reporting bias. Poor-quality SRs will be excluded from our review.

For newly identified RCTs, we will use the Cochrane Collaboration’s risk of bias (ROB) tool [30], which categorizes biases by six domains (selection bias, performance bias, detection bias, attrition bias, reporting bias, and other bias) and makes a summary assessment. For each item, a summary rating (high, low, or unclear ROB) is assigned along with a succinct free-text description to support the rating.

## Data collection and extraction

Data from published fair- or good-quality reviews and newly identified RCTs will be abstracted into a customized database by one reviewer and overread by a second reviewer. The data abstraction form will be agreed upon and piloted by all study team members in advance of full data extraction. Disagreements will be resolved by consensus or by obtaining a third reviewer’s opinion when consensus cannot be reached. Data elements abstracted will include descriptors to characterize the type of study, study population (including number participants, mean age, race/ethnicity, clinical indication, baseline severity), intervention (including dose and how delivered), comparator, outcomes reported (including prespecified subgroup analyses), study quality, moderator effect or meta-regression analyses, and author conclusions. In particular, we will collect the following information important to the occurrence of VMS: the specific definition of perimenopause used by authors, inclusion of patients with a history of breast cancer and/or those receiving any chemotherapeutic agents, and inclusion or exclusion of women who underwent surgical menopause. Description of study populations will be taken as reported in the SRs and will be verified in the original RCTs only if there is concern for accuracy.

## Data synthesis

**Fig. 1:**
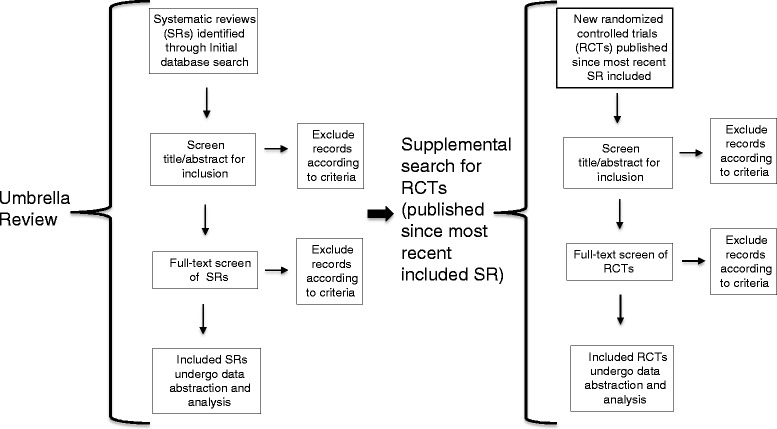
Literature flow diagram

We will illustrate the article flow and number of included studies in a literature flow diagram (Fig. 1). We will group the SRs and RCTs by intervention.

## Data synthesis from SRs

We will prioritize SRs that are the highest quality, most current, and most relevant to the study question [22]. Relevance will be assessed using the PICOTS (population, intervention, comparator, outcome, timing, setting) framework along with the search date, review methods, and completeness of reporting. For each intervention, we will provide tables or graphical displays to describe the studies included, study quality, and treatment effects. If informative, we will display a treatment network, describing the number and types of interventions evaluated. We will report intervention effects separately for inactive and active comparators and plan to describe effects using a “forest top plot” [17] and in table form. We will examine SRs for relevant subgroup analyses, including concurrent use of hormone replacement therapy, effects in women with and without breast cancer, perimenopausal and postmenopausal women, and women with surgical versus natural menopause. If multiple high-quality, relevant reviews are included on a single intervention, we will provide a matrix comparing which studies were included in the reviews.

## Data synthesis from RCTs

Although umbrella reviews do not typically search for new primary studies, our review incorporates this step in order to identify important new data. Provided the volume of newly identified primary studies is manageable, we plan to incorporate these studies into our assessment. We will search the bibliographies of all the SRs and compare the results of the RCTs with the conclusions drawn by the SRs. We will also clearly identify data derived from existing SRs and data abstracted from newly identified RCTs as part of this umbrella review.

## Meta-analysis

Newly identified RCTs will be summarized qualitatively and, if indicated, quantitatively (i.e., meta-analysis). However, no clear rules exist for when a new quantitative synthesis needs to be conducted. We will consider the number of new studies, the sample size, and the strength of evidence domains [29] as a framework for determining whether a new quantitative synthesis is indicated. If new primary studies are likely to change the judgments about the strength of evidence, we will conduct an updated meta-analysis. If the new studies are consistent with prior syntheses and likely will not change the conclusion of the review, then we will not complete an updated meta-analysis.

## Narrative summary

In addition, we will narratively describe the major findings and conclusions from the existing reviews. We will identify evidence gaps by documenting clinical indications for which there is conflicting evidence across identified reviews or where reviews concluded that the existing evidence base is insufficient to reach firm conclusions. We also will document topic areas for which SRs exist but which did not identify relevant RCTs.

## Subgroup analysis

When possible, we plan to extract data from eligible SRs to perform subgroup analysis and further clarify the relationship with specific interventions and VMS. Specific subgroups of interest include women with a history of breast cancer and women with a history of surgical menopause.

## Grading the evidence

The strength of evidence for the key question will be assessed using the approach described in AHRQ’s *Methods Guide for Effectiveness and Comparative Effectiveness Reviews* [18]. In brief, this approach requires assessment of four domains: risk of bias, consistency, directness, and precision. Additional domains are to be used when appropriate, including coherence, dose-response association, impact of plausible residual confounders, strength of association (magnitude of effect), and publication bias. These domains will be considered qualitatively, and a summary rating will be assigned after discussion by two reviewers as high, moderate, or low strength of evidence. In some cases, high, moderate, or low ratings will be impossible or imprudent to make. In these situations, a grade of insufficient will be assigned.

## Discussion

Our review is designed as an umbrella review supplemented by an additional search for eligible RCTs published after the relevant SRs. We aim to synthesize the existing evidence on the use of nonpharmacologic, nonherbal interventions to manage bothersome VMS in perimenopausal and postmenopausal women. Our findings will help clinicians and women experiencing impaired quality of life from VMS to identify potentially effective nonpharmacological treatments as well as quantify the effect of such options. If possible, we hope to provide additional clarity on differential effects of nonpharmacologic treatments among those with and without breast cancer and those who underwent natural versus surgical menopause.

## Limitations

We acknowledge that there are potentially other effective nonpharmacologic interventions that may benefit women who are experiencing bothersome VMS that will not be included in our review based on our current protocol. The choice of included interventions was determined by expert opinion from the field and those of highest relevance to practice within the VHA. Selected interventions are meant to reflect currently used and widely available treatments while keeping the scope of this review feasible. We limited our search to SRs and RCTs that were in English, which may have excluded important studies conducted in different languages. Of note, some SRs may have included trials that were not published in English.

## Ethics approval and consent to participate

Not applicable.

## Consent for publication

Not applicable.

## Additional files

Additional file 1: Completed PRISMA-P checklist for this protocol. (DOCX 32 kb) Additional file 2: Sample review form used to assess quality of SRs. (DOCX 33 kb)

## Abbreviations

AHRQ: Agency for Healthcare Research and Quality
HRQOL: health-related quality of life
RCT: randomized controlled trials
SR: systematic reviews
VHA: Veterans Health Administration
