# Erratum to: Nonpharmacologic, nonherbal management of menopause-associated vasomotor symptoms: an umbrella systematic review (protocol)

**DOI:** 10.1186/s13643-016-0248-y

**Published:** 2016-04-25

**Authors:** Karen M. Goldstein, Jennifer R. McDuffie, Megan Shepherd-Banigan, Deanna Befus, Remy R. Coeytaux, Megan G. Van Noord, Adam P. Goode, Varsha Masilamani, Soheir Adam, Avishek Nagi, John W. Williams

**Affiliations:** Center for Health Services Research in Primary Care, Durham Veterans Affairs Medical Center, Durham, NC USA; Department of Medicine, Division of General Internal Medicine, Duke University Medical Center, 411 W. Chapel Hill Street; Suite 500, Durham, NC 27701 USA; Duke University School of Nursing, Durham, NC USA; Duke Clinical Research Institute, Durham, NC USA; Duke University Medical Center Library, Durham, NC USA; Department of Physical Therapy, Duke University Medical Center, Durham, NC USA; Department of Medicine, Duke University Medical Center, Durham, NC USA

After publication of this work [1], the authors noticed that one source of funding was missing in the funding section. The correct information is given below:

## References

[1] Goldstein KM, McDuffie JR, Shepherd-Banigan M, Befus D, Coeytaux RR, Van Noord MG, Goode AP, Masilamani V, Adam S, Nagi A, Williams JW (2016). Nonpharmacologic, nonherbal management of menopause-associated vasomotor symptoms: an umbrella systematic review (protocol). Systematic Reviews.

