# Supplementary material for: Nonpharmacologic, nonherbal management of menopause-associated vasomotor symptoms: an umbrella systematic review (protocol)
Source: Syst Rev. 2016 Apr 7;5:56. doi: 10.1186/s13643-016-0232-6 (PMC4823860; doi:10.1186/s13643-016-0232-6)
Supplement: Additional file 2: — Sample review form used to assess quality of SRs. (DOCX 33 kb) [file 13643_2016_232_MOESM2_ESM.docx]

**Additional File: Quality Assessment for Systematic Reviews**

General instructions: The purpose of this rating tool is to evaluate the scientific quality of systematic reviews (SRs). It is not intended to measure the literary quality, importance, relevance, originality, or other attributes of SRs.

**Step 1: Determine whether it is a SR. SRs are studies that (1) include an explicit and adequate search, (2) apply prespecified eligibility criteria, and (3) consider the quality of included studies or risk of bias assessment, and/or describe plans to synthesize or attempt to synthesize findings quantitatively and/or qualitatively.**

**Step 2: For SRs, grade each of the criteria listed below as “Yes,” “No,” “Can’t tell” or “Not Applicable.” Factors to consider when making an assessment are listed under each criterion. For each domain, summarize key methods, level of concern overall (low, high, unclear), and rationale for concerns.**

**STUDY ELIGIBILITY CRITERIA**

1. **Was an ‘a priori’ design provided?**

The research question and inclusion criteria should be established before the conduct of the review and the review should adhere to pre-defined objectives and eligibility criteria.

*Note*: *Need to refer to a protocol, ethics approval, or pre-determined/a priori published research objectives to score a “yes.”*
[] Yes [] No [] Can’t tell [] N/A

Comment:

1. **Were study eligibility criteria clearly specified?***Note: Criteria should be sufficiently detailed to allow replication of study*[] Yes [] No [] Can’t tell [] N/A

Comment:

1. **Were restrictions in eligibility criteria appropriate?** Restrictions based on study characteristics (e.g., date, sample size, study quality, outcomes measured) and based on sources of information (e.g., publication status or format, language) should be appropriate?
   [] Yes [] No [] Can’t tell [] N/A

Comment

Summarize key methods related to Eligibility:

Summarize concerns (low/high/unclear) and your rationale regarding specification of study eligibility criteria:

**IDENTIFICATION AND SELECTION OF STUDIES**

1. **Was a comprehensive literature search performed?**

At least 2 electronic sources should be searched and electronic searches should be supplemented by consulting: reference lists from prior reviews, textbooks, or included studies; specialized registries (e.g., Cochrane registries); or queries to experts in the field.

*Note: If at least 2 sources + one supplementary strategy used, select “yes”; grey literature search counts as supplementary*

[] Yes [] No [] Can’t tell [] N/A
Comment:

1. **Were the terms and structure of the search strategy likely to retrieve as many eligible studies as possible?***Note: Search methods should describe: search date, databases used, and search terms (Key words and/or MESH terms must be stated and where feasible the search strategy should be provided).*[] Yes [] No [] Can’t tell [] N/A
   Comment:
2. **Were restrictions based on date, publication format, or language appropriate?**[] Yes [] No [] Can’t tell [] N/A
   Comment:
3. **Was selection bias avoided?**

The review reports the number of studies identified through searches, the numbers excluded, and gives appropriate reasons for excluding – based on explicit inclusion/exclusion criteria. Two or more raters made inclusion/exclusion decisions.

[] Yes [] No [] Can’t tell [] N/A
Comment:

Summarize key methods related to Study Selection:

Summarize concerns (low, high, unclear) and rationale regarding Study Selection:

**DATA COLLECTION AND STUDY APPRAISAL**

1. **Was there duplicate study selection and data extraction?**

Did two or more investigators abstract data, and assess study quality – either independently or with one rater over-reading the first raters result?

Was an appropriate method used to resolve disagreements (e.g., a consensus procedure)?

[] Yes [] No [] Can’t tell [] N/A

Comment:

1. **Were the characteristics of the included studies provided?**

In an aggregated form such as a table, data from the original studies should be provided on the participants, interventions and outcomes. The ranges of characteristics in all the studies analyzed (e.g., age, race, sex, relevant socioeconomic data, disease status, duration, severity or other diseases) should be reported in sufficient detail to allow the review authors and readers to interpret the results.

*Note: Acceptable if not in table format as long as they are described as above.*

[] Yes [] No [] Can’t tell [] N/A
Comment:

1. **Was the scientific quality of the included studies assessed and documented?**

A priori methods of assessment should be provided and criteria used to assess study quality specified in enough detail to permit replication.

*Note: Can include use of a quality scoring tool or checklist, e.g., Jadad scale, risk of bias, sensitivity analysis, etc., or a description of quality items, with some kind of result for EACH study (“low” or “high” is fine, as long as it is clear which studies scored “low” and which scored “high”; a summary score/range for all studies is not acceptable).*

[] Yes [] No [] Can’t tell [] N/A
Comment:

Summarize key methods related to data collection/ROB:

Summarize concerns regarding data collection/ROB:

**SYNTHESIS AND FINDINGS**

1. **Was the scientific quality of the included studies used appropriately in formulating conclusions?**

The results of the methodological rigor and scientific quality should be considered in the analysis (e.g. subgroup analyses) and the conclusions of the review, and explicitly stated in formulating recommendations. *Note: Might say something such as “the results should be interpreted with caution due to poor quality of included studies.” Cannot score “yes” for this question if scored “no” for question 10.*

[] Yes [] No [] Can’t tell [] N/A

1. **Were the methods used to combine the findings of studies appropriate?**

For pooled results, the synthesis should be appropriate given the nature and similarity of included studies (conceptual homogeneity), and an accepted quantitative method of pooling should be used (i.e., more than simple addition; e.g., random-effects or fixed-effect model). If only qualitative analyses are completed, the study should describe the reasons that quantitative analyses were not completed.

[] Yes [] No [] Can’t tell [] N/A
Comment:

1. **Was between-study variation (heterogeneity) minimal or addressed in the synthesis?**For pooled results, a qualitative and quantitative assessment of homogeneity (Cochran’s Q and/or I^2^) should be performed.

   Note: Indicate “yes” if they explain that they cannot pool because of heterogeneity/variability between interventions.

   [] Yes [] No [] Can’t tell [] N/A
   Comment:

1. **Was the likelihood of publication bias assessed?**

Publication bias tested using funnel plots, test statistics (e.g., Egger’s regression test), and/or search of trials registry for unpublished studies.
 *Note: If no test values, clinical trials.gov search, or funnel plot included, score “no”. Score “yes” if mentions that publication bias could not be assessed because there were fewer than 10 included studies*.

[] Yes [] No [] Can’t tell [] N/A
Comment:

1. **Are the stated conclusions supported by the data presented?**Were the conclusions made by the author(s) supported by the data and/or analyses reported in the systematic review? Conclusions should address limitations of the systematic review and limitations of the primary studies. Conclusions should consider relevance of the included studies to the research question.
   [] Yes [] No [] Can’t tell [] N/A

Summarize key methods related to synthesis:

Summarize concerns regarding synthesis:

**OTHER**

1. **Was the conflict of interest stated?**Potential sources of support should be clearly acknowledged in both the systematic review and the included studies.
   *Note:* To get a “yes,” must indicate source of funding or support for the systematic review AND for each of the included studies
   [] Yes [] No [] Can’t tell [] N/A
   Comment:

**Step 3: Rate the overall quality of the SR as “Good,” “Fair,” or “Poor” using the guidance below and summarize major reasons for rating in the comments box.**

**Good** = After considering items 1-15, item 15 is rated “Yes” with no important limitations. This means that few of the items 1-14 are rated “No,” and none of the limitations are thought to decrease the validity of the conclusions. If items 3, 4, 7, 9, 10, 11 or 12 are rated “no”, then the review is likely to have major flaws

**Fair** = After considering items 1-15, item 15 is rated “Yes,” but with at least some important limitations. This means that enough of the items 1-15 are rated “No” to introduce some uncertainty about the validity of the conclusions.

**Poor** = After considering items 1-15, item 15 is rated “No.” This means that several of items 1-15 are rated “No,” introducing serious uncertainty about the validity of the conclusions.

Overall rating comments:

**Concerns regarding specifications of eligibility criteria**

| Low concern | Considerable effort has been made to clearly specify the review question and objectives, and to pre-specify and justify appropriate and detailed eligibility criteria that have been adhered to during the review |
| --- | --- |
| High concern | Studies that would have been important and relevant to answering the review question are likely to have been excluded from the review, either due to the lack of pre-specified objectives and eligibility criteria, or because inappropriate restrictions were imposed or studies that are not appropriate for addressing the review question have been included. |
| Unclear concern | Insufficient information is reported to make a judgement about risk of bias. |

**Concerns regarding methods used to identify and/or select studies**

| Low concern | Given the review question and eligibility criteria as assessed in Domain 1, a substantial effort has been made to identify as many relevant studies as possible through a variety of search methods using a sensitive and appropriate search strategy and steps were taken to minimise bias and errors when selecting studies for inclusion. |
| --- | --- |
| High concern | Some eligible studies are likely to be missing from the review. |
| Unclear concern | There is insufficient information reported to make a judgement on risk of bias. |

**Concerns regarding methods used to collect data and appraise studies**

| Low concern | Given the studies included in the review as assessed in domain 2, risk of bias was assessed using appropriate criteria, data extraction and risk of bias assessment involved two reviewers, and relevant study characteristics and results were extracted |
| --- | --- |
| High concern | Some bias may have been introduced through the data collection or risk of bias assessment processes. |
| Unclear concern | There is insufficient information reported to inform a judgement on risk of bias. |



**Concerns regarding methods used to synthesize results**

| Low concern | The synthesis is unlikely to produce biased results, because any limitations in the data were overcome, or the findings were so convincing that the limitations would have little impact |
| --- | --- |
| High concern | The synthesis is likely to produce biased results, because (i) potential biases were ignored (within and/or across studies), (ii) important between-study variation was not accounted for; (iii) there were important inadequacies in the methodology; or (iv) findings are incompletely reported in a way that raises concerns. |
| Unclear concern | There is insufficient information reported to make a judgement on risk of bias. |

**Methods adapted from:**

1. Marinopoulos SS, Dorman T, Ratanawongsa N, Wilson LM, Ashar BH, Magaziner JL, et al. Effectiveness of continuing medical education. Evid Rep Technol Assess (Full Rep). 2007;(149):1-69.
2. Moher D, Cook DJ, Eastwood S, Olkin I, Rennie D, Stroup DF. Improving the quality of reports of meta-analyses of randomised controlled trials: the QUOROM statement. Quality of Reporting of Meta-analyses. Lancet. 1999;354(9193):1896-1900.
3. Shea BJ, Grimshaw JM, Wells GA, Boers M, Andersson N, Hamel C, et al. Development of AMSTAR: a measurement tool to assess the methodological quality of systematic reviews. BMC Med Res Methodol. 2007;7(1):1-7.
4. Whiting P, Savovic J, Higgins JP, Caldwell DM, Reeves BC, Shea B, et al. ROBIS: A new tool to assess risk of bias in systematic reviews was developed. J Clin Epidemiol. 2015.
